# Application of a multifunctional chemotherapy infusion device for reducing antineoplastic drug extravasation

**DOI:** 10.3389/fonc.2025.1539389

**Published:** 2025-03-06

**Authors:** Li-Hua Yang, Li-Ping Liu, Fa-Ying Jiang, Feng-Zhu Huang, Chun-Fen Xie, Xue-Qin Lin, Pan Wang, Xiu-Li Feng

**Affiliations:** ^1^ Department of Neurology, Panyu Branch, The Second Affiliated Hospital, Guangzhou Medical University, Guangzhou, China; ^2^ Department of Outpatient, Panyu Branch, The Second Affiliated Hospital, Guangzhou Medical University, Guangzhou, China; ^3^ Blood Purification Centre, Panyu Branch, The Second Affiliated Hospital, Guangzhou Medical University, Guangzhou, China; ^4^ Management Committee, Panyu Branch, The Second Affiliated Hospital, Guangzhou Medical University, Guangzhou, China

**Keywords:** antineoplastic drugs, drug extravasation, injury, infusion device, intravenous infusion, multifunctional chemotherapy

## Abstract

**Objective:**

This study aimed to address the challenges associated with antineoplastic drug extravasation during intravenous administration, through the development of a novel chemotherapy infusion device. A secondary objective was to mitigate associated risks to healthcare personnel, patients, caregivers and the environment.

**Methods:**

A water-soluble fluorescent solution was used as a surrogate for antineoplastic chemotherapy agents to assess the potential for drug extravasation and the associated risks of occupational exposure during intravenous administration. The investigation identified risks related to drug extravasation, which informed the development of the novel infusion device.

**Results:**

In experiment 1, conventional methods for replacing infusion bags resulted in drug extravasation during the second bag change across all procedures conducted by 9 operators. Specifically, extravasation was observed in 81 out of 90 procedures. In experiment 2, the newly designed multifunctional chemotherapy infusion device, which requires each infusion bag to be punctured only once, was used. Under these conditions, the same 9 operators performed 90 procedures, with extravasation occurring in only 2 instances.

**Conclusion:**

The multifunctional chemotherapy infusion device facilitates the efficient administration of intravenous chemotherapy while addressing the issue of drug extravasation associated with traditional infusion devices during the delivery of antineoplastic drugs. This device effectively reduces the risk of occupational injuries among healthcare workers, reduces harm to patients and their caregivers, and mitigates environmental contamination.

## 1 Introduction

Cancer represents a significant global public health challenge, with its incidence steadily increasing, as reported in the 2020 Global Cancer Statistics (1). Since the first application of nitrogen mustard in 1942 for the treatment of lymphoma and leukemia, chemotherapy has remained a cornerstone of cancer treatment. The synthesis and clinical success of agents like cyclophosphamide and fluorouracil in 1957 further advanced the field, drawing widespread attention to chemotherapy as a key approach in improving cancer survival rates. Antineoplastic drugs (ADs) have played a key role in enhancing patient outcomes; however, their therapeutic efficacy is accompanied by adverse effects on normal cells (2).

ADs can enter the human body through multiple routes, including the respiratory tract, skin contact, the digestive system, and needlestick injuries (3). Their teratogenic, carcinogenic, and mutagenic risks have been extensively documented by organizations such as the National Institute for Occupational Safety and Health (NIOSH) (4–17).

Extensive research has been conducted globally on the preparation and administration of ADs, particularly focusing on the issue of extravasation and associated protective measures (15–23). Studies have demonstrated that intravenous administration of ADs contributes to surface contamination in healthcare environments, posing occupational hazards to medical personnel, including physicians, pharmacists, pharmacy technicians, nurses, auxiliary staff, and cleaning personnel. The U.S. Occupational Safety and Health Administration (OSHA) has published the NIOSH List of Hazardous Drugs in Healthcare Settings, which provides detailed guidelines for healthcare workers. These guidelines recommend the use of personal protective equipment (PPE), the establishment of safe operating procedures, regular training and education for healthcare workers exposed to hazardous drugs, and the use of closed-system transfer devices (CSTD) to minimize drug leakage and environmental contamination. In Europe, various measures have been adopted by different countries for managing occupational exposure to ADs. However, a unified regulation or standard has not yet been established. The Directive (EU) 2024/869 aims to protect workers from health and safety risks associated with carcinogenic, mutagenic, or reprotoxic substances (CMR) in the workplace, including the substitution of hazardous substances, the use of closed systems, reduction of exposure, PPE, and hygiene measures, among others. In China, regulations and standards such as the Law on the Prevention and Control of Occupational Diseases and the Regulations on the Management of Medical Waste have been established. These regulations clearly stipulate that medical institutions must implement protective measures, provide training, and offer education to healthcare workers regarding occupational exposure to hazardous substances. Mei Xiaohong et al. (24) underscored two fundamental principles for occupational safety: ① minimizing unnecessary exposure to ADs and ② reducing environmental contamination caused by ADs. However, in some low- and middle-income countries, occupational protection measures against exposure to ADs may not yet have received adequate attention due to limited resources.

Despite extensive research, specific stages of intravenous infusion during which healthcare workers experience occupational exposure to Ads have not been explicitly identified. Furthermore, there is a lack of effective recommendations and measures targeting extravasation at these stages. Research on the harm caused by AD extravasation to other patients in the same ward and to caregivers present in the environment remain limited.

This study focuses on the design of a multifunctional chemotherapy infusion device aimed at mitigating extravasation during the administration of ADs. The device is intended to significantly reduce unnecessary occupational exposure to ADs and minimize contamination within the healthcare environment.

## 2 Methods

### 2.1 General data

Clinical nurses who hold a nursing practice certificate, demonstrate proficiency in intravenous infusion skills, have experience in the use of ADs, possess knowledge of protective measures against exposure to ADs, and have skills in managing occupational exposure were selected as participants, and categorized based on their years of clinical experience: <5 years, ≥5 years but <10 years, and ≥10 years, with three nurses in each group. All participants underwent standardized training in intravenous infusion techniques and infusion bag replacement procedures which was provided by specialist intravenous therapy nurses. Participation in the experimental procedures was limited to those who successfully passed the competency assessments.

A colorless, transparent, water-soluble fluorescent solution (prepared by dissolving 0.1 g of a fluorescent agent in 100 mL of 0.9% sodium chloride, hereinafter referred to as the “fluorescent solution”) was used as a substitute for ADs during the experiments. The packaging integrity of the fluorescent solution was verified under 365 nm ultraviolet light prior to the experiments to confirm the absence of external contamination.

Sterile, certified intravenous infusion devices equipped with plastic needles were used for all procedures. The experiments were carried out in an unoccupied three-bed hospital ward to replicate real clinical conditions. To facilitate the detection of fluorescent solution extravasation, the door and windows of the ward were kept closed, and blackout curtains were used to maintain a dark environment.

Drug extravasation was defined as the presence of any fluorescent solution outside the infusion bag or tubing.

The study protocol was approved by the Clinical Research and Application Ethics Committee of the Second Affiliated Hospital of Guangzhou Medical University (No.: 2023-ks-23) and followed the Declaration of Helsinki and its subsequent amendments. All nurses participating in the study signed an informed consent form after fully understanding the purpose, methods, potential risks, and benefits of the study. The study did not involve operations with harmful substances, and all experimental procedures were conducted by nurses who had undergone professional training. All waste generated during the study was disposed of in accordance with environmental and safety regulations to avoid harm to the environment and public health.

### 2.2 Experimental methods

#### 2.2.1 Grouping

Nine nurses were categorized into three groups according to their years of clinical experience. Standardized training in intravenous infusion techniques and infusion bag replacement procedures was provided by specialist intravenous therapy nurses. Inclusion in the experimental procedures was limited to participants who successfully completed and passed the competency assessments.

Group 1: Nurses with < 5 years of clinical experience (3 participants).

Group 2: Nurses with ≥ 5 years but < 10 years of clinical experience (3 participants).

Group 3: Nurses with ≥ 10 years of clinical experience (3 participants).

The sample size of 3 nurses per group was determined based on practical constraints, including the availability of participants. To enhance the reliability of the data, each nurse was observed 10 times, resulting in a total of 30 observations per group. We acknowledge that the small number of nurses per group limits the generalizability of the findings, and the study should be interpreted as an exploratory investigation.

#### 2.2.2 Procedure

**Figure 1:**
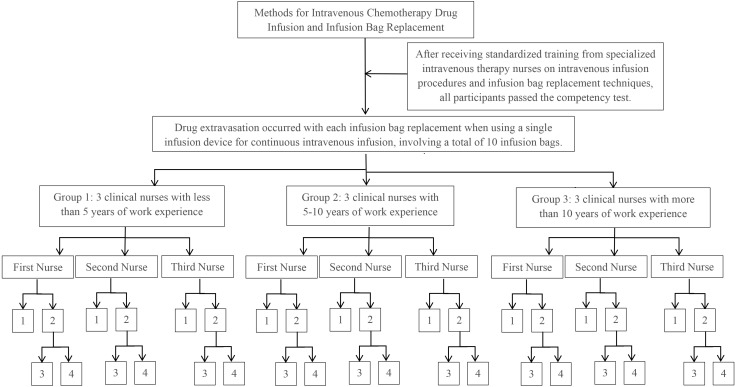
Experimental procedure for experiment 1. 1: No occurrence of drug extravasation. 2: Occurrence of drug extravasation. 3: The number of infusion bag replacements during which drug extravasation occurred. 4: The extent of drug extravasation after completing the replacement of 10.

Experiment 1: Each participant conducted 10 consecutive infusion bag replacements. If drug extravasation occurred during the replacement process, the procedure was continued until all 10 replacements were completed. A total of 90 procedures were performed by the 9 participants (see **Figure 1**).

The incidence and extent of drug extravasation during each procedure were documented, and the extravasation rates were calculated.

**Figure 2:**
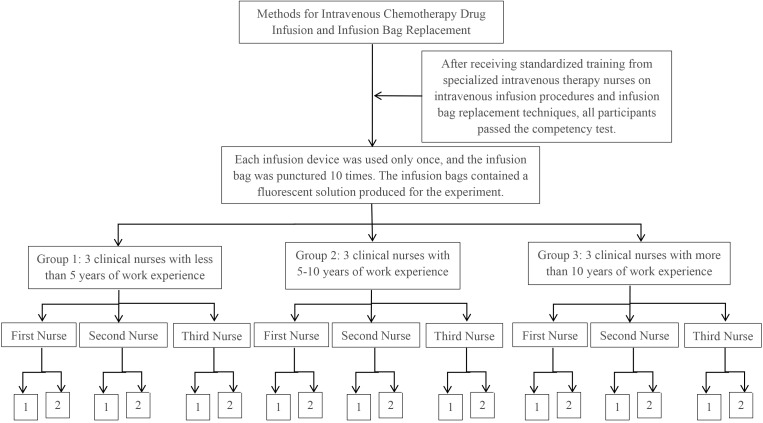
Experimental procedure for experiment 2. 1: No occurrence of drug extravasation. 2: Number of occurrences of drug extravasation.

Experiment 2: Each participant conducted 10 infusion bag replacements, ensuring that the puncture needle of each infusion device was used for a single puncture to connect to a prepared fluorescent solution bag. Drug extravasation was recorded for each procedure, with a total of 90 procedures conducted by the 9 participants (see **Figure 2**).

### 2.3 Data statistics and analysis

#### 2.3.1 Data statistics

Occurrences of drug extravasation during experiments 1 and 2 were recorded at the following sites: puncture points and taper bases of the infusion bags. The observed sites on the left hand included the wrist, palm, dorsum of the hand, dorsal side of fingers (thumb, index, middle, ring, and little fingers), and palmar side of fingers (thumb, index, middle, ring, and little fingers). The sites on the right hand included the wrist, palm, dorsum of the hand, dorsal side of fingers (thumb, index, middle, ring, and little fingers), and palmar side of fingers (thumb, index, middle, ring, and little fingers).

The extent of drug extravasation was classified into four levels: None (0): No fluorescent solution detected at any observation site. Minor (1): Fluorescent solution detected at the puncture point and/or the taper base of the infusion bag. Moderate (2): Fluorescent solution detected at the puncture point and/or the taper base of the infusion bag, in addition to any area on either hand. Large (3): Fluorescent solution detected at the puncture point and/or the taper base of the infusion bag, along with any area on both hands.

Drug extravasation was recorded as binary data (0 = no extravasation, 1 = extravasation), while the extent of extravasation was documented as ordinal data (0 = none, 1 = minor, 2 = moderate, 3 = large).

Results of experiment 1:

Group 1: Out of 30 bag replacements, 3 procedures involved no extravasation, while 27 procedures involved extravasation (5 minor, 0 moderate, 22 large).

Group 2: Out of 30 bag replacements, 3 procedures involved no extravasation, while 27 involved extravasation (3 minor, 2 moderate, 22 large).

Group 3: Of the 30 bag replacements, 3 procedures involved no extravasation, while 27 involved extravasation (2 minor, 1 moderate, 24 large).

Details are presented in **Table 1**.

**Table 1:** Results from experiment 1.

Objects of Study	Infusion Bag Replacement Count	Years of Work Experience	Extravasation Occurrence (0 = No, 1 = Yes)	Extravasation Range (0 = No, 1 = Minor, 2 = Moderate, 3 = Large)
①	1	Nurses with work experience < 5 years	0	0
	2		1	1
	3		1	1
	4		1	1
	5		1	3
	6		1	3
	7		1	3
	8		1	3
	9		1	3
	10		1	3
②	1	Nurses with work experience < 5 years	0	0
	2		1	1
	3		1	3
	4		1	3
	5		1	3
	6		1	3
	7		1	3
	8		1	3
	9		1	3
	10		1	3
③	1	Nurses with work experience < 5 years	0	0
	2		1	1
	3		1	3
	4		1	3
	5		1	3
	6		1	3
	7		1	3
	8		1	3
	9		1	3
	10		1	3
④	1	Nurses with work experience ≥5 but < 10 years	0	0
	2		1	1
	3		1	2
	4		1	3
	5		1	3
	6		1	3
	7		1	3
	8		1	3
	9		1	3
	10		1	3
⑤	1	Nurses with work experience ≥5 but < 10 years	0	0
	2		1	1
	3		1	2
	4		1	3
	5		1	3
	6		1	3
	7		1	3
	8		1	3
	9		1	3
	10		1	3
⑥	1	Nurses with work experience ≥5 but < 10 years	0	0
	2		1	1
	3		1	3
	4		1	3
	5		1	3
	6		1	3
	7		1	3
	8		1	3
	9		1	3
	10		1	3
⑦	1	Nurses with work experience ≥ 10 years	0	0
	2		1	3
	3		1	3
	4		1	3
	5		1	3
	6		1	3
	7		1	3
	8		1	3
	9		1	3
	10		1	3
⑧	1	Nurses with work experience ≥ 10 years	0	0
	2		1	1
	3		1	3
	4		1	3
	5		1	3
	6		1	3
	7		1	3
	8		1	3
	9		1	3
	10		1	3
⑨	1	Nurses with work experience ≥ 10 years	0	0
	2		1	1
	3		1	2
	4		1	3
	5		1	3
	6		1	3
	7		1	3
	8		1	3
	9		1	3
	10		1	3
Extravasation Occurrence:	0: No extravasation; 1: Extravasation occurs (fluorescent agent found outside the infusion bag or infusion device tube).			
Extravasation Range:	Minor: Fluorescent solution detected at the puncture point and/or the taper base of the infusion bag. Moderate: Fluorescent solution detected at the puncture point and/or the infusion bag, in addition to any area on either hand. Large: Fluorescent solution detected at the puncture point and/or the taper base of the infusion bag, along with any area on both hands.			

Results of experiment 2:

Group 1: Out of 30 bag replacements, 29 procedures involved no extravasation, while 1 procedure involved minor extravasation.

Group 2: Among 30 bag replacements, 29 procedures involved no extravasation, with 1 procedure involving minor extravasation.

Group 3: All 30 bag replacements were conducted without any extravasation.

**Figure 3:**
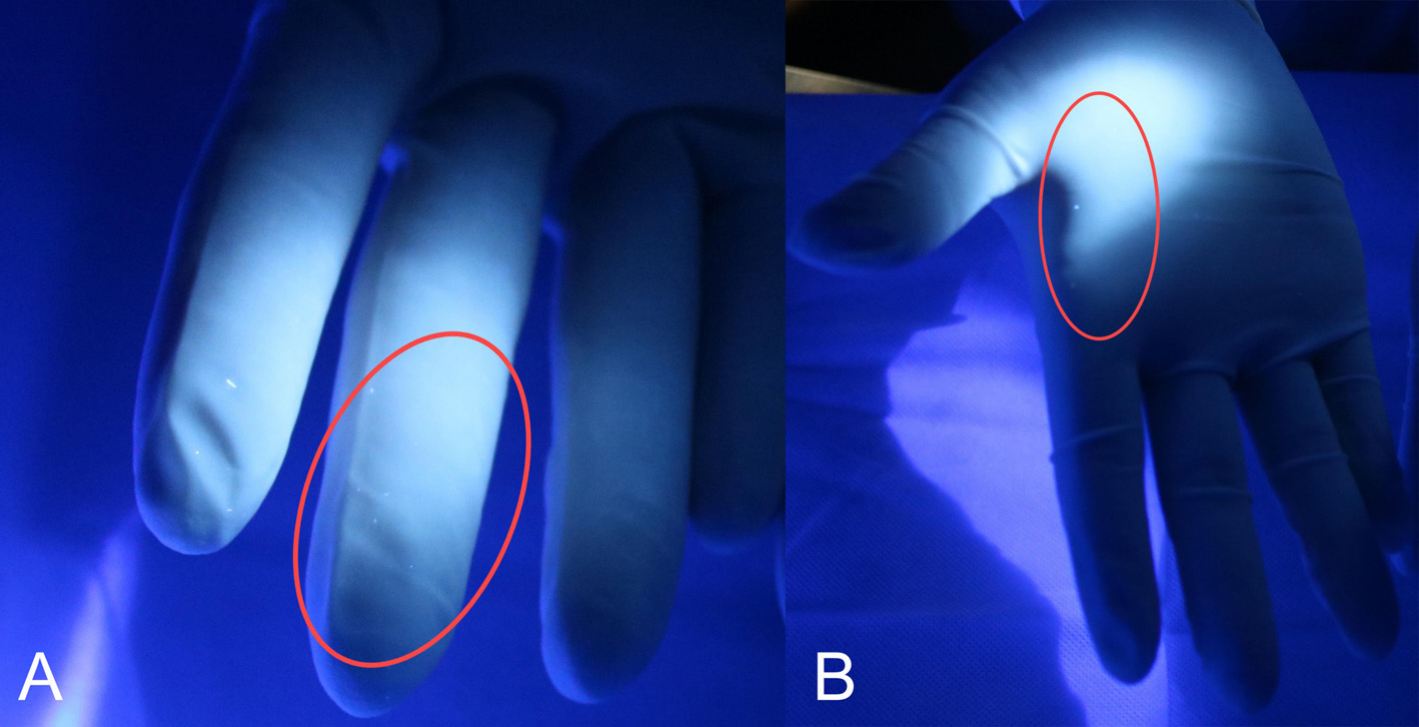
Drug extravasation observed during the replacement of the infusion device. The fluorescent agent on the fingers **(A)** and palm **(B)** of the right hand detected by a 365-nm UV light after the replacement of the infusion bag during the experimental operation.

Details are provided in **Table 2**, and observations of drug extravasation are depicted in **Figure 3**.

**Table 2:** Results from experiment 2.

Objects of Study	Infusion Bag Replacement Count	Years of Work Experience	Extravasation Occurrence (0 = No, 1 = Yes)	Extravasation Range (0 = No, 1 = Minor, 2 = Moderate, 3 = Large)
①	1	Nurses with work experience < 5 years	0	0
	2		0	0
	3		0	0
	4		0	0
	5		0	0
	6		1	1
	7		0	0
	8		0	0
	9		0	0
	10		0	0
②	1	Nurses with work experience < 5 years	0	0
	2		0	0
	3		0	0
	4		0	0
	5		0	0
	6		0	0
	7		0	0
	8		0	0
	9		0	0
	10		0	0
③	1	Nurses with work experience < 5 years	0	0
	2		0	0
	3		0	0
	4		0	0
	5		0	0
	6		0	0
	7		0	0
	8		0	0
	9		0	0
	10		0	0
④	1	Nurses with work experience ≥5 but < 10 years	0	0
	2		0	0
	3		0	0
	4		0	0
	5		0	0
	6		0	0
	7		0	0
	8		0	0
	9		0	0
	10		0	0
⑤	1	Nurses with work experience ≥5 but < 10 years	0	0
	2		0	0
	3		0	0
	4		1	1
	5		0	0
	6		0	0
	7		0	0
	8		0	0
	9		0	0
	10		0	0
⑥	1	Nurses with work experience ≥5 but < 10 years	0	0
	2		0	0
	3		0	0
	4		0	0
	5		0	0
	6		0	0
	7		0	0
	8		0	0
	9		0	0
	10		0	0
⑦	1	Nurses with work experience ≥ 10 years	0	0
	2		0	0
	3		0	0
	4		0	0
	5		0	0
	6		0	0
	7		0	0
	8		0	0
	9		0	0
	10		0	0
⑧	1	Nurses with work experience ≥ 10 years	0	0
	2		0	0
	3		0	0
	4		0	0
	5		0	0
	6		0	0
	7		0	0
	8		0	0
	9		0	0
	10		0	0
⑨	1	Nurses with work experience ≥ 10 years	0	0
	2		0	0
	3		0	0
	4		0	0
	5		0	0
	6		0	0
	7		0	0
	8		0	0
	9		0	0
	10		0	0
Extravasation Occurrence:	0: No extravasation; 1: Extravasation occurs (fluorescent agent found outside the infusion bag or infusion device tube).			
Extravasation Range:	Minor: Fluorescent solution detected at the puncture point and/or the taper base of the infusion bag. Moderate: Fluorescent solution detected at the puncture point and/or the infusion bag, in addition to any area on either hand. Large: Fluorescent solution detected at the puncture point and/or the taper base of the infusion bag, along with any area on both hands.			

#### 2.3.2 Data analysis

Data analysis was conducted using SPSS 27.0 software. The occurrence and extent of exposure were treated as quantitative variables. For data following a normal distribution, results are presented as mean ± standard deviation (SD), and statistical comparisons were conducted using independent-sample or paired-sample t-tests. For non-normally distributed data, results are expressed as median ± interquartile range (IQR), and non-parametric tests were applied. A *P*-value < 0.05 was considered statistically significant.

## 3 Results

### 3.1 General data of two groups

The analysis indicated that work experience had no statistically significant impact on the occurrence of occupational exposure to ADs during intravenous bag replacement (*p* = 0.953) or the extent of exposure (*p* = 0.995). Detailed results are provided in **Table 3**.

**Table 3:** Comparison of exposure outcomes among nurse groups with varying levels of work experience.

Group	Exposure Count	Exposure Range
Nurses with work experience < 5 years	5±9	11±25
Nurses with work experience ≥ 5 but < 10 years	5±9	12.5±24
Nurses with work experience ≥ 10 years	4.5±9	12±26
H	0.097	0.09
P	0.953	0.995

### 3.2 Analysis of occupational exposure results in 9 clinical nurses

The use of the multifunctional chemotherapy infusion device significantly decreased both the incidence of occupational exposure to ADs (*p* = 0.005) and the extent of exposure (*p* < 0.001) during intravenous bag replacement. Detailed results are presented in **Table 4**.

**Table 4:** Comparison of exposure outcomes across groups using different infusion methods.

Group	Replacement Count	Exposure Count	Exposure Range
Traditional Infusion Device	90	9±0	25±1
Novel Infusion Device	90	0±1	0±1
z/t		-2.810	37.806
p		0.005	<0.001

### 3.3 Design of the novel infusion device

**Figure 4:**
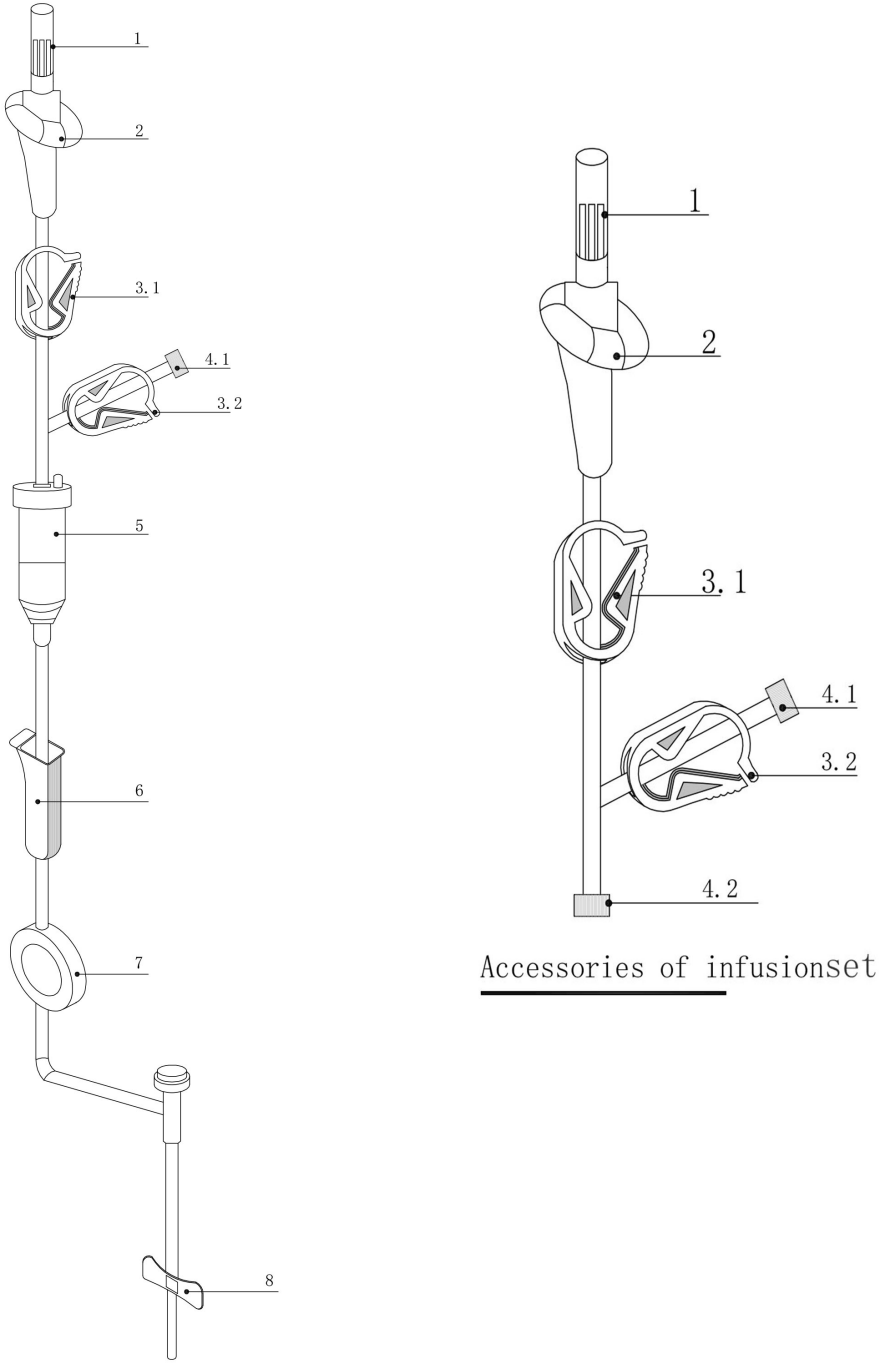
Infusion device and its accessories. 1: Infusion puncture needle; 2: Air inlet; 3.1/3.2: Clamps; 4.1: Male Luer taper; 4.2: Female Luer taper; 5: Drip chamber; 6: Flow regulator; 7: Filter; 8: Intravenous infusion needle.

Data analysis from experiments 1 and 2 demonstrated that using each infusion needle for a single puncture and maintaining a continuous connection throughout the infusion process, regardless of the number of infusion bags, significantly reduced the occurrence and extent of occupational exposure to ADs during intravenous bag replacement. Based on these findings, a multifunctional chemotherapy infusion device was designed with the following features:

The device consists of a disposable system incorporating 1+n (n ≥ 0) parallel infusion lines. Its components include one complete infusion device (comprising a puncture needle, air inlet, infusion tube, clamp, Luer connector, drip chamber, flow regulator, filter, and intravenous infusion needle) and n (n ≥ 0) additional infusion accessories (each comprising a puncture needle, air inlet, infusion tube, clamp, and Luer taper). The structural components of the device and its accessories are depicted in **Figure 4**, where 4.1 represents the male Luer taper, and 4.2 represents the female Luer taper.

Unused infusion accessories are configured with the clamp at 3.2 in a closed position, and the female Luer taper at 4.2 capped with the male Luer taper (see **Figure 4**). If no additional infusion channels are required, the clamp at 3.2 on the final infusion channel remains closed, and the male Luer taper at 4.1 is capped with the female Luer taper. When additional infusion channels are necessary, the male Luer taper at 4.1 on the final infusion channel is uncapped and connected to the female Luer taper at 4.2 of the infusion accessories, thereby establishing a new infusion channel.

This multifunctional chemotherapy infusion device represents a closed infusion system designed to accommodate additional drug infusion channels as required for treatment.

When using the multifunctional chemotherapy infusion set, only the clamp at 3.1 (see **Figure 4**) on the active infusion channel is opened. For infusion channels of accessories connected to the main infusion device, both the clamp at 3.1 on the accessory and the clamp at 3.2 on the main infusion line are opened. Upon completing the drug infusion, the clamp at 3.1 on the active channel is closed, followed by the opening of the 3.2 clamp along with the 3.1 clamp of the subsequent infusion accessory. This ensures that only one infusion channel remains active at any given time, effectively preventing unintended drug mixing unless explicitly indicated by medical orders. For mixed drug infusions, the corresponding 3.1 and 3.2 clamps on the selected channels are opened, while all other clamps remain closed.

This innovative multifunctional chemotherapy infusion device addresses the limitations associated with traditional infusion systems and demonstrates potential for clinical application.

## 4 Discussion

Research conducted by Ness demonstrated that healthcare professionals handling Ads, even with the appropriate use of personal protective equipment (PPE), experience higher levels of oxidative stress compared to those who do not handle these agents (25). Similarly, studies by Sottani and others, using biological and environmental biomarkers, confirmed that healthcare personnel such as physicians, pharmacists, pharmacy technicians, nurses, auxiliary nurses, and cleaning staff are at risk of occupational exposure to the harmful effects of ADs (20, 26, 27). In response to these findings, several recommendations have been proposed: (1) implementing risk management strategies to minimize and maintain exposure at the lowest possible levels; (2) conducting biological and environmental monitoring to ensure the long-term efficacy of preventive measures; and (3) incorporating considerations for the use of gloves when handling ADs, either directly or indirectly, as well as addressing contamination risks associated with specific tasks. These measures should also be incorporated into training and information dissemination for healthcare workers at risk of exposure. However, Yu noted that while exposure to ADs cannot be completely avoided, no fundamental solutions to this issue were proposed (28).

Research by Forges emphasized that the use of safe infusion devices significantly reduces the risk of occupational exposure to ADs for nurses (29). However, the study also confirmed that occupational exposure occurs during the disconnection of infusion bags, for which no solutions were provided.

To address the challenges highlighted in previous studies, the objective of this study was to develop solutions to reduce the biological and environmental hazards associated with the intravenous infusion of ADs.

The findings indicate that when conventional infusion devices were used for intravenous therapy, all operators in the experimental groups experienced drug extravasation during the second infusion bag replacement. In contrast, the use of the newly designed chemotherapy infusion device resulted in only two instances of extravasation, both of which were attributed to improper operation: the first incident involved a nurse from Group 1 (with less than 5 years of work experience). The extravasation occurred during the sixth puncture of the infusion bag containing fluorescent solution with the infusion needle. The cause was that the nurse did not fully insert the infusion needle into the bag before withdrawing it and then attempted to puncture the bag again. This operation is equivalent to one of the operations between the 2nd and 10th attempts in Experiment 1. The second incident involved a nurse from Group 2 (with work experience between 5 and 10 years). The extravasation occurred during the 4th puncture of the infusion bag containing fluorescent solution. The cause was that the nurse did not accurately target the center of the infusion port on the bag. Instead, the needle punctured the edge of the port, causing an obstruction and subsequent drug extravasation. Statistical analysis revealed no significant differences in the extravasation rate and the extent of occupational exposure between experimental groups, indicating that the experience of nurses has no significant impact on the extravasation rate. This may be related to the fact that, prior to this experiment, all nurses involved were uniformly trained by a specialist nurse in intravenous therapy in the operations of intravenous infusion and infusion bag replacement, and they all passed the assessment. Leso (30) also pointed out that training is one of the effective measures to improve occupational protection. These results demonstrate that training and maintaining a single puncture connection between the needle of the infusion device and the infusion bag, without subsequent disconnection, significantly reduces both the rate of extravasation and the extent of occupational exposure to ADs during bag replacement. Thus, the multifunctional chemotherapy infusion device developed in this study effectively prevents drug extravasation throughout the infusion process.

Intravenous therapy remains one of the most frequently performed nursing procedures in clinical practice. A national survey conducted in 2020 examining the use of intravenous infusions in inpatient settings at secondary-level and above general hospitals reported that 86.10% of hospitalized patients received intravenous infusions. The average daily use per hospital bed was 2.69 bottles or bags, with a mean daily volume of 556.56 mL per bed. Utilization rates for specific types of intravenous infusions were as follows: Chinese medicine injections (18.10%), antimicrobial injections (39.58%), proton pump inhibitor injections (19.11%), antiemetic injections (9.35%), and parenteral nutrition formulations (12.41%) (31).

The multifunctional chemotherapy infusion device developed in this study is a closed infusion system designed to accommodate the addition of drug channels based on specific treatment regimens. The components of the device and its accessories are connected using Luer tapers. Once the infusion needle is connected to a medication bag, it remains connected until the infusion is complete, after which both the medication bag and the infusion device are discarded. This closed design eliminates the need for repeated needle disconnections and reconnections, a practice commonly associated with traditional infusion methods. Such repetitive handling in conventional systems increases the risk of chemotherapy drug extravasation, environmental contamination, and occupational exposure to hazardous agents.

The multifunctional chemotherapy infusion device features a simple structure, ease of operation, and adaptability, allowing the addition or removal of infusion channels as required for different treatment regimens. It effectively addresses the issue of chemotherapy drug extravasation associated with infusion bag replacement during the infusion process. In addition to its application in chemotherapy, the device is suitable for administering general medications and facilitates alternating infusions of chemotherapy drugs and general medications, thus functioning as a versatile and multi-purpose tool for intravenous therapy. Considering usability and cost: Currently, the price of infusion sets on the market ranges from 2 to 6 RMB per unit. A comprehensive assessment indicates that the production cost of the infusion set accessories is approximately 0.5 RMB per unit. When more than one bag of infusion drugs is used, each additional bag increases the consumable cost by about 0.5 RMB. Although the cost of consumables for infusion operations has increased, the authors believe that compared to the potential harm to healthcare workers, other individuals in the infusion environment, and the environment itself, the benefits of using a multifunctional chemotherapy infusion set are increased.

This study has several limitations due to restricted experimental conditions:

1. The sample size is small, with a total of 90 procedures performed by 9 participants in a single center, which may limit the generalizability of the results. This study is just a starting point, and we plan to conduct multicenter studies and increase the sample size in future research to enhance the external validity of our findings.
2. Experimental Solution: Due to ethical considerations, AD solutions could not be used in the extravasation experiments. A colorless, transparent, water-soluble fluorescent solution (concentration: 0.1 g of fluorescent agent dissolved in 100 mL of 0.9% sodium chloride) was used as a substitute. The fluorescent solution is safer for healthcare workers, the environment, and other individuals in the environment. The fluorescent solution serves as a visualization tool, enabling us to monitor the distribution of the spilled drug in real time and provide intuitive experimental data. However, it was not possible to confirm whether the extravasation rate of the fluorescent solution accurately reflects that of AD solutions during the infusion process, thereby limiting the applicability of the results. Furthermore, it remains uncertain whether changes in the concentration of the fluorescent solution or its use in alternative solutions (e.g., 5% glucose, 10% glucose, 10% sodium chloride-glucose, or 0.5% sodium bicarbonate solutions) would yield extravasation rates comparable to those observed with the fluorescent solution used in the study. Under the premise of ensuring the safety of the operators, we will gradually introduce actual ADs in future studies to verify the stages of extravasation during the intravenous use of ADs.
3. Result Observation: The observations of fluorescent agent extravasation were conducted visually using 365 nm ultraviolet light. No precise instruments were used to detect or quantify the extravasation at each site. Consequently, the volume of extravasated solution at each location could not be determined, limiting the accuracy of the data.
4. Extravasation Range Statistics: The experiment recorded fluorescence appearances at specific sites, including the puncture points of infusion bags, taper bases of infusion bags, and various areas of the left and right hands (e.g., wrist, palm, dorsum, and dorsal and palmar sides of fingers). However, other areas, such as the body, bed unit, bedside cabinet, bedside chair, and the floor adjacent to the bed of the simulated patient, were not observed or recorded. The occurrence of extravasation was assessed solely based on the presence of fluorescence at the observed sites, without precise quantification of the affected areas. As a result, the extent of extravasation reported does not accurately represent the actual amount of extravasation.

Despite the limitations posed by the use of a substitute experimental solution and the absence of precise detection instruments, this experiment confirmed the occurrence of drug extravasation during the process of changing infusion bags in intravenous infusion. Furthermore, the multifunctional chemotherapy infusion device developed in this study effectively reduces drug extravasation during intravenous infusion treatments.

## 5 Conclusion

The novel multifunctional chemotherapy infusion device developed in this study offers an effective solution to the challenges of intravenous chemotherapy administration, particularly in mitigating the risk of drug extravasation associated with traditional infusion devices during the delivery of ADs. This device effectively reduces occupational risks to healthcare workers, reduces harm to patients and caregivers, and reduces environmental contamination. Subsequently, while ensuring the safety of the operators, we will progressively incorporate actual ADs to elucidate the specific stages at which extravasation occurs during intravenous administration. We will compare the extravasation incidence rate observed when using traditional infusion sets with real ADs to the rate documented in this study using fluorescent solution. In addition to its primary application in the infusion of ADs, the device offers flexibility for use in the intravenous administration of general medications, including the alternating infusion of chemotherapy and general drugs. This versatility enhances its adaptability in diverse clinical settings, making it a practical solution for preventing drug extravasation during intravenous therapy. However, the present study mainly focuses on short-term results, and future studies should evaluate the long-term performance and reliability of the multifunctional infusion device, including its durability, ease of use over extended periods, and potential for user fatigue or error.

Given its demonstrated effectiveness in improving safety and efficacy, the implementation of this multifunctional chemotherapy infusion device in clinical practice is strongly recommended.

## Data Availability

The original contributions presented in the study are included in the article/supplementary material. Further inquiries can be directed to the corresponding authors.
